# Evaluation of oncological outcomes in patients with oral cavity cancer treated in a low-volume hospital^☆^^☆☆^

**DOI:** 10.1016/j.bjorl.2025.101621

**Published:** 2025-06-04

**Authors:** Sara de Souza Bettioli, Agnaldo José Graciano, Ana Lavratti Borga, Carlos Augusto Fischer

**Affiliations:** Hospital Municipal São José, Departamento de Cirurgia de Cabeça e Pescoço, Joinville, SC, Brazil

**Keywords:** Oral cavity, Tongue, Floor of mouth e lower lip, Malignant neoplasm

## Abstract

**Objective:**

To evaluate the results of OCC patients treated in low-volume regional hospitals.

**Methods:**

This is a retrospective longitudinal study conducted with patients diagnosed with OCC and operated on in a low-volume hospital between January 2003 and December 2018.

**Results:**

174 patients with OCC were treated at the institution ‒ an average of 11 patients/year. The most common tumor location was the tongue (48.2%), followed by the lip (18.2%). Squamous cell carcinomas were the most frequent (94.7% of patients). Adjuvant radiotherapy and chemotherapy were performed in 46.7% and 31.9% of patients, respectively. Almost 21% of patients had some postoperative complication. Specific survival of 62.6% and global survival of 58.2% after 3-years were similar to the results reported in high-volume centers. Disease-free survival was 45.8% in the same period.

**Conclusion:**

Low-volume hospitals qualified for oncological treatments can present outcomes similar to those of high-volume centers, and are thus a regional option for patients with OCC.

**Level of evidence:**

Level III.

## Highlights

• The overall and disease-free survival was similar to those in high volume centers.
• The complications rate were not different as described in high volume centers.
• Oncological low volume centers presents with a equal local and regional recurrences rates.

## Introduction

Oral Cavity Cancer (OCC) corresponds to approximately 30% of Head and Neck (H&N) neoplasms, and is among the 10 most prevalent types of tumors in the world, with a ratio of 2 men to 1 woman.^1^, ^2^, ^3^ Despite the decreased incidence of this disease in recent years, mainly associated with a reduction in risk factors such as smoking and alcohol consumption, around 400,000 new cases still occur worldwide per year, mainly in developing countries.^2^, ^4^ In Brazil, the estimate for 2020 was 14,200 new cases, and the state of Santa Catarina had one of the highest incidence rates, ranging from 11.2 to 19.9 cases per 100,000 inhabitants.^5^

Squamous Cell Carcinoma (SCC) is the most common histological type of this neoplasm, occurring in up to 90% of cases, with the remaining 10% distributed among sarcomas, lymphomas, salivary cell tumors, among others.^6^

Surgical resection with adequate margin is the most commonly indicated initial procedure for patients with OCC, and is commonly associated with treatment of regional lymph nodes, followed by adjuvant radiotherapy and chemotherapy, depending on tumor type and pathological stage.^7^

In general, a large proportion of OCC patients are diagnosed when tumors are in advanced stages, and thus have a worse prognosis even when these patients undergo combined treatments: surgery, chemotherapy, and radiotherapy. It is estimated that the overall 5-year survival rate for OCC patients varies between 50% and 65%.^1^, ^6^, ^8^ Factors such as delayed onset of symptoms and difficulty in reaching a definitive diagnosis and in access to treatment are among the causes of worse outcomes; however, when these tumors are diagnosed early, survival rates can reach 80%, and this is the best prognostic factor.^6^, ^8^

Some studies have suggested that better oncological outcomes are obtained at lower treatment costs when OCC patients are treated in tertiary and quaternary centers, with ample availability of resources for multidisciplinary treatment.^2^, ^9^ In these institutions, the 5-year survival rate can reach 61% of cases, while for patients treated in non-quaternary hospitals, this rate is approximately 40%.^10^, ^11^

This study aimed to assess the oncological outcomes of OCC patients treated at a low-volume, non-quaternary, regional institution and to compare them with those reported in high-volume centers.

## Materials and methods

This is a longitudinal retrospective study that evaluated data from the medical records of OCC patients operated on in a non-quaternary regional reference institution between January 2002 and December 2018. This study was approved by the Research Ethics Committee of the aforementioned Institution under protocol no. CAAE: 45752821.1.0000.5362.

The following data were collected: demographic, including age and gender; clinical and pathological staging (pTNM); measures of disease-free survival (time elapsed between initial treatment and local, regional, or distant tumor recurrence) during follow-up; 3-year overall survival and specific disease. Data on complications in the early postoperative period until hospital discharge, and late complications requiring readmission were also analyzed. Comparative analyses were performed between patients whose tumor primary site was the lip (lower and upper) vs. other whose primary sites were the oral cavity (tongue, floor of mouth, retromolar trigone, hard palate, and buccal mucosa).

The collected data were processed using the SPSS.20, Minitab.16 and Excel Office 2010 software. The quantitative variables were analyzed through application of the nonparametric Mann-Whitney test.

The Equality of Two Ratios test was used to compare the proportion of responses to two specific variables and/or their levels of statistical significance. Survival analyses were performed using the Kaplan-Meier estimate.

## Results

Data from the medical records of 213 patients diagnosed with OCC treated at the aforementioned Institution were analyzed. Of these, 174 patients were initially included in the study, but four of them had their follow-up censored during treatment. On average, 11 patients per year underwent surgical treatment of the oral cavity between 2003 and 2018.

Most patients were men (*n* = 140; 82.3%), while women represented 17.6% of cases, both with a mean age of 56.9-years (ranging from 25- to 104-years), with a little over 98% of these identified as Caucasian.

The most common tumor primary site was the tongue (48.2%), followed by the lip (18.2%) (Table 1). Advanced staging III and IV were the most frequent, present in 54.7% of cases. Elective or therapeutic cervical lymphadenectomy was performed in 71% of the patients, and cervical metastases were confirmed upon pathological examination in 72 (32.9%) of these cases, with the majority (40.2%) staged as N1 (Table 2).

**Table 1:** Distribution among primary sites of OCC.

Primary site	*n*	%
Tongue	82	48.20%
Lip	31	18.20%
Floor of mouth	29	17.10%
Retromolar trigone	15	8.80%
Hard palate	6	3.50%
Upper lip	2	2.40%

**Table 2:** Distribution of OCC staging among patients.

Staging		*n*	%
T	Tis + T1	48	28.4%
	T2	49	29.0%
	T3 + T4	72	42.6%
N	N0	96	57.1%
	N1	29	17.3%
	N2a	12	7.1%
	N2b	23	13.7%
	N2c	4	2.4%
	N3b	4	2.4%
CS	0‒I‒II	77	45.3%
	III‒IV	93	54.7%

The use of flaps to reconstruct the surgical defect was necessary in 61.8% of cases, and the pectoralis flap was the most common (57%) (Table 3).

**Table 3:** Type of flap in descending order of frequency.

Type of flap	*n*	%
Pectoralis	57	57%
Locally advanced	26	26%
Submental	13	13%
Microsurgical	4	4%

Adjuvant radiotherapy was indicated in 46.7% of patients, while concomitant chemotherapy and radiotherapy was used in 31.9% of cases.

The rate of complications in the initial postoperative period was 21.2%, with suture dehiscence and hematoma as the most frequently observed, occurring in 7.3% and 6.1% of patients, respectively (Table 4).

**Table 4:** Postoperative complications.

Complication	*n*	%
Suture dehiscence	12	7.3%
Hematoma	11	6.7%
Wound infection	10	6.1%
Salivary fistula	8	4.8%
Immediate reoperation	8	4.8%
Immediate PO death	2	1.2%

PO, Postoperative.

Mean follow-up time (time elapsed between surgery and the last visit) ranged from 36- to 48.6-months, and average disease-free time (time elapsed between recurrence and surgery) was 28.8-months.

**Fig. 1:**
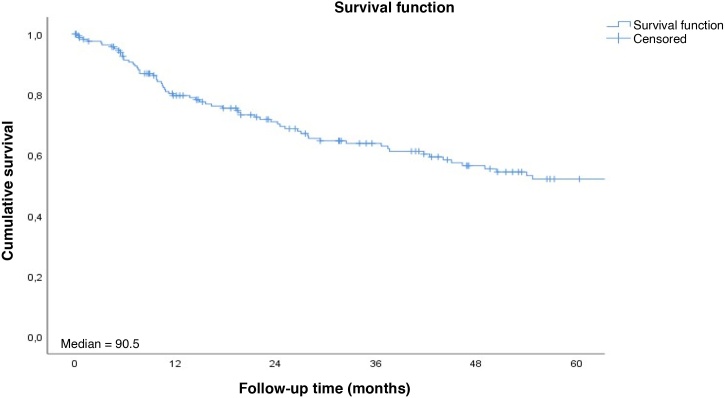
Overall survival rate to follow-up time curve. Cumulative survival; Median = 90.5; Survival function; Survival function; Censored; Follow-up time (months).

**Fig. 2:**
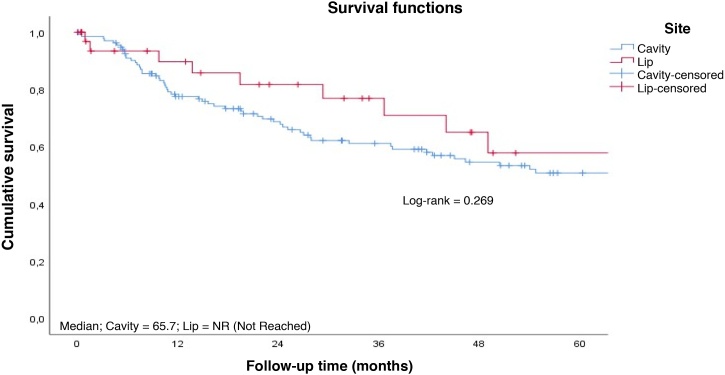
Survival rate to follow-up time curve by tumor site. Cumulative survival; Median; Cavity = 65.7; Lip = NR (Not Reached); Survival functions; Log-rank = 0.269; Site; Cavity; Lip; Cavity-censored; Lip-censored; Follow-up time (months).

**Fig. 3:**
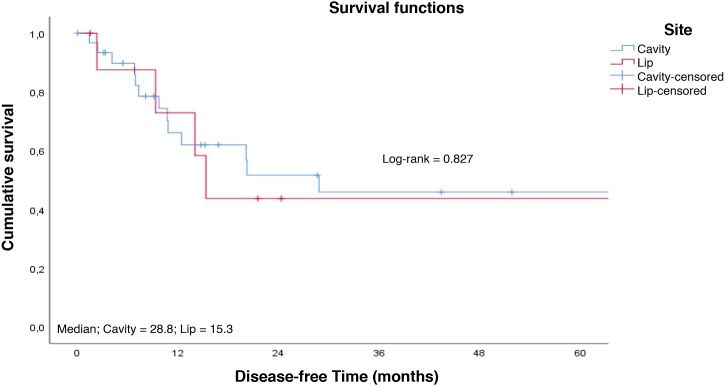
Survival rate to disease-free time curve by tumor site. Cumulative survival; Median; Cavity = 28.8; Lip = 15.3; Survival functions; Log-rank = 0.827; Site; Cavity; Lip; Cavity-censored; Lip-censored; Disease-free Time (months).

The 3-year specific and overall survival rates were 62.6% and 58.2%, respectively (Fig. 1). Patients with SCC of the lip presented longer survival compared with those with tumor in other sites of the oral cavity altogether (Figs. 2‒ 3).

**Fig. 4:**
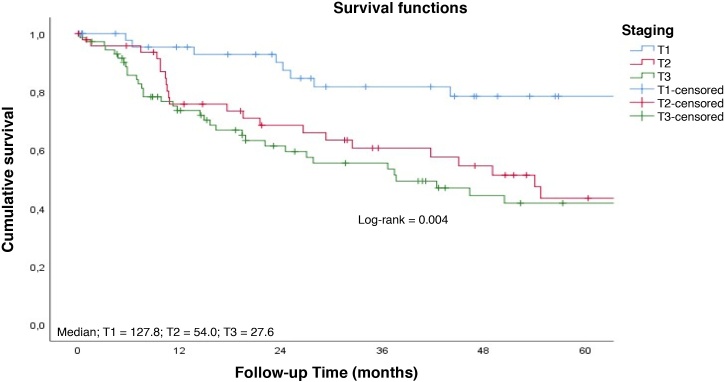
Survival rate to follow-up time curve by T staging. Cumulative survival; Median; T1 = 127.8; T2 = 54.0; T3 = 27.6; Survival functions; Log-rank = 0.004; Staging; T1; T2; T3; Cavity-censored; T1-censored; T2-censored; T3-censored; Follow-up Time (months).

**Fig. 5:**
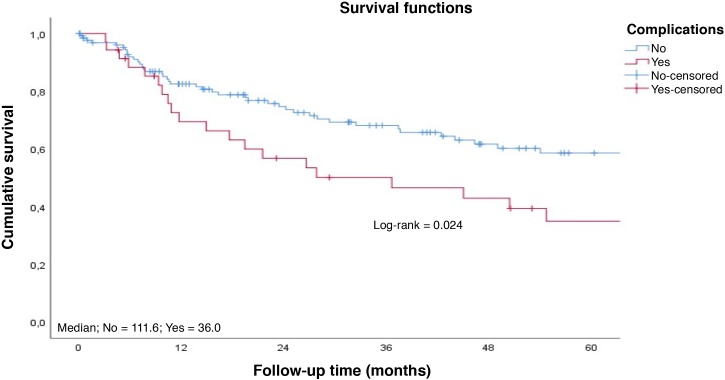
Survival rate to follow-up time curve by complications. Cumulative survival; Median; No = 111.6; Yes = 36.0; Survival functions; Log-rank = 0.024; Complications; No; Yes; No-censored; Yes-censored; Follow-up Time (months).

**Fig. 6:**
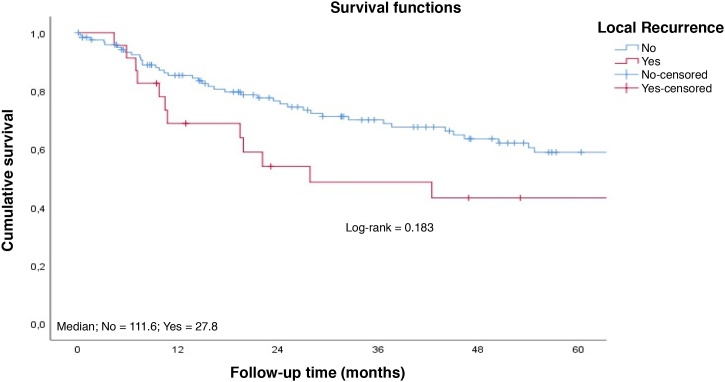
Survival rate to follow-up time curve by local recurrence. Cumulative survival; Median; No = 111.6; Yes = 27.8; Survival functions; Log-rank = 0.183; Local Recurrence; No; Yes; No-censored; Yes-censored; Follow-up Time (months).

**Fig. 7:**
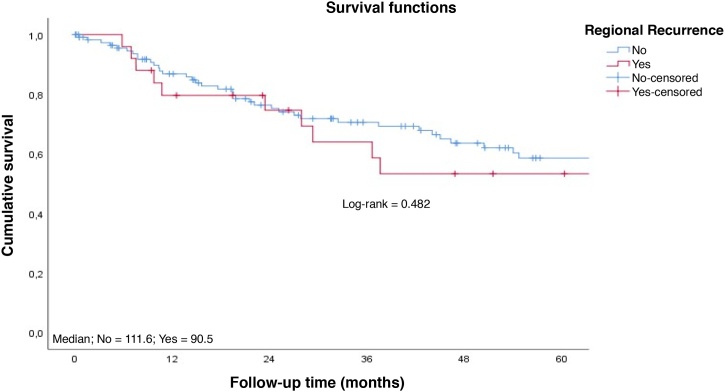
Survival rate to follow-up time curve by regional recurrence. Cumulative survival; Median; No = 111.6; Yes = 90.5; Survival functions; Log-rank = 0.482; Regional Recurrence; No; Yes; No-censored; Yes-censored; Follow-up Time (months).

Patients with early tumors with no postoperative complications had a longer long-term survival (Figs. 4‒ 5). Occurrence of local and/or regional recurrence was associated with decreased survival time (Figs. 6‒ 7).

Approximately 32.4% of the patients died after 30-months on average, whereas only 38.2% were alive and free of disease after 60-months.

## Discussion

The vast majority of OCC cases (82%) occurred in men, as a result of increased contact with risk factors such as smoking and alcohol consumption. However, in recent years, the incidence of OCC has increased in women because of the increase in cases of Human Papillomavirus (HPV) and their greater contact with other known risk factors.^4^

The patients were presented at diagnosis with a mean age similar to those found in developed countries such as Japan (65-years) and in non-quaternary hospitals in Brazil (60-years).^12^

Without much variation when compared with other epidemiological studies, the tongue and floor of mouth were the most common tumor sites after the lip. According to a North American study conducted with more than 20,000 patients, tongue cancer has a higher 5-year mortality risk than those in other sites.^13^

The worst prognostic factor for OCC patients is late diagnosis, which according to a Canadian literature review, is based on three components: patient’s delay in seeking assistance, waiting to be referred to a specialized professional, and time elapsed before starting treatment.^8^ This study showed that 54.7% of the patients were diagnosed when their tumors were at advanced stages III and IV. In a retrospective study including cases treated between 1973 and 2014, Cheraghlou et al.^14^ also observed that 50.2% of patients were presented with advanced OCC. When compared with similar retrospective national studies, the same tendency for late diagnoses is observed, suggesting that patients have difficulty in quickly accessing specialists and initiating early treatment.^15^

As a consequence of diagnosis at advanced disease stages, cervical lymphadenectomy was needed in about 70% of the cases, with confirmed metastasis in 32.9% of the histopathological exams.

For tumors at advanced stages (III and IV) and with unfavorable risk factors, postsurgical adjuvant treatments are needed, and a large part of the sample was submitted to adjuvant radiotherapy (46.7%) and chemotherapy (31.9%).

The rate of postoperative complications was significantly lower compared with those reported by similar studies, as observed in a Brazilian retrospective study involving 159 patients, in which 47.3% of the sample had surgical site infection and 53.7% had suture dehiscence ‒ values much higher than those found in the present study (6.1% for wound infection and 7.3% for suture dehiscence). This same study reported that microsurgical flaps would have a much lower risk of orocutaneous fistulae; however, due to the difficult access to microsurgery in our service, this type of flap was performed in only 4% of patients.^16^

The average number of patients operated on per year was 11, which characterizes this hospital a low-volume hospital. According to an Asian study, from 22 cases/year, a hospital can be considered a high-volume hospital.^17^ The 3-year survival rate (58.2%) was similar to that observed in high-volume hospitals, as in a study conducted with 58,295 patients in which this rate was 61.5%.^10^ However, in general, it is observed that patients treated in high- volume institutions have a higher 5-year survival (51.8%) than those treated in low-volume institutions (45.5%).^18^ It is believed that part of these results can be justified by the fact that high-volume hospitals have more specialized professionals, more multidisciplinary care in the postoperative period, and more intensive care resources.^19^

Patients with early diagnoses show significant improvement in survival time, as demonstrated in a retrospective study conducted with over 16,000 patients: those diagnosed at earlier stages had a 3-year survival rate of 92.2%, whereas the rate for those with late diagnosis was 70.3% for the same period studied.^14^

A higher mean mortality rate was found compared with those of high-volume hospitals. According to a Brazilian retrospective study, the 1-year mortality rate for OCC patients was 18.96%, and the risk increases according to age, and has higher prevalence in men from the North and Northeast regions of the country.^20^

## Conclusion

Compared with other reference hospitals and centers, similar percentages were observed in relation to 3-year survival rate, late diagnosis, and epidemiological profile of patients. However, a lower rate of postoperative complications and a higher mean overall mortality were observed. Therefore, it is observed that low-volume hospitals qualified for oncological treatments can present results similar to those of high-volume centers, and are thus a regional option for OCC patients.

## Conflicts of interest

The authors declare no conflicts of interest.
